# Transcranial Electrical Neuromodulation Based on the Reciprocity Principle

**DOI:** 10.3389/fpsyt.2016.00087

**Published:** 2016-05-27

**Authors:** Mariano Fernández-Corazza, Sergei Turovets, Phan Luu, Erik Anderson, Don Tucker

**Affiliations:** ^1^NeuroInformatics Center, University of Oregon, Eugene, OR, USA; ^2^LEICI Instituto de Investigaciones en Electrónica, Control y Procesamiento de Señales, Universidad Nacional de La Plata (UNLP), CONICET, La Plata, Argentina; ^3^Electrical Geodesics Inc., Eugene, OR, USA; ^4^Department of Psychology, University of Oregon, Eugene, OR, USA

**Keywords:** transcranial electrical stimulation, non-invasive neuromodulation, transcranial direct current stimulation, reciprocity principle, high-density electrode arrays

## Abstract

A key challenge in multi-electrode transcranial electrical stimulation (TES) or transcranial direct current stimulation (tDCS) is to find a current injection pattern that delivers the necessary current density at a target and minimizes it in the rest of the head, which is mathematically modeled as an optimization problem. Such an optimization with the Least Squares (LS) or Linearly Constrained Minimum Variance (LCMV) algorithms is generally computationally expensive and requires multiple independent current sources. Based on the reciprocity principle in electroencephalography (EEG) and TES, it could be possible to find the optimal TES patterns quickly whenever the solution of the forward EEG problem is available for a brain region of interest. Here, we investigate the reciprocity principle as a guideline for finding optimal current injection patterns in TES that comply with safety constraints. We define four different trial cortical targets in a detailed seven-tissue finite element head model, and analyze the performance of the reciprocity family of TES methods in terms of electrode density, targeting error, focality, intensity, and directionality using the LS and LCMV solutions as the reference standards. It is found that the reciprocity algorithms show good performance comparable to the LCMV and LS solutions. Comparing the 128 and 256 electrode cases, we found that use of greater electrode density improves focality, directionality, and intensity parameters. The results show that reciprocity principle can be used to quickly determine optimal current injection patterns in TES and help to simplify TES protocols that are consistent with hardware and software availability and with safety constraints.

## Introduction

Transcranial electrical stimulation (TES) is also known as transcranial direct current stimulation (tDCS) and transcranial alternating current stimulation (tACS), depending on the nature of the applied current. Because the current levels are typically small (1–2 mA) and do not actually stimulate neuronal firing, the method is also termed transcranial electrical neuromodulation (TEN). Even without stimulating neuronal firing, TES or TEN applications are capable to modify cortical excitability (1, 2) as well as brain rhythms and networks (3, 4). In comparison with transcranial magnetic stimulation (TMS), TES is a portable, cost-effective, and easy-to-use tool. TES is an emerging therapy for the treatment of neuropsychiatric conditions such as depression (5), Parkinson’s disease (6), anxiety, and chronic pain (7). Research has also demonstrated that TES can be a valuable therapeutic tool in epilepsy (8), stroke rehabilitation (9), and other neurological and psychiatric conditions (10). It has also been proposed to enhance cognitive skills such as memory or learning (11, 12). This technique may become eventually an alternative for psychoactive drugs, as it ideally does not affect the entire brain indiscriminately, and it has minimal adverse side effects. The requirement for specific targeting of neural regions of interest (ROI) is to use a methodology minimizing, as much as possible, current applied to non-target areas.

Despite these recent advances, there are ongoing debates on the clinical effectiveness of TES (13–15) addressing many issues to be still resolved, in particular, substantial inter-subject response variability. As described for instance in Ref. (16), only about 36% of the participants showed the canonical pattern of anodal-facilitatory/cathodal-inhibitory after-effects that are typically assumed in the literature. Furthermore, the evidence of a non-linear relation between current dosage and measured after-effects (17) implies that consistency of treatment may be highly sensitive to dosage precision. Because current flow cannot be fully focused, but rather follows the path of least resistance through the head tissues, an accurate model of electrode positions and head conductivity is required. In addition, because current is likely to have different effects when aligned with the neuronal columns (normal to the cortical surface) than when crossing them (tangential flow) (18, 19), it is important to model the individual’s cortical geometry with cortical surface extraction from individual anatomical MRI (20), in order to compute the components of the induced current density that are normal to cortex versus those that are tangential. Moreover, there is an increasing interest in moving beyond the current use of two large sponge electrodes, such as with the local “high-definition” pattern of one source electrode surrounded by four sinks (21) or all-head dense electrode arrays, to increase the precision of TES. Improving the specification of current density at the gyri-precise target for each individual subject, thereby computing the effective dosage individually, may be important to account for the considerable variability that is observed across individuals (5, 16, 22). In this regard, the anatomically faithful subject-specific human head modeling in TES is becoming to play an increasingly more important role to facilitate and set the scene of the ultimate testing of TES clinical effectiveness in the future clinical trials.

Several studies have been conducted to compute the delivered dose to ROI and to optimize the electrode shape and size as well as the total electrode number and configuration. The estimation of the delivered dose to the cortical ROI is usually done by means of finite element (FE) or finite difference (FD) simulations of the current densities in detailed three-dimensional models of the head (23, 24). FE simulations showed that high current density may occur in unexpected regions, due to the relatively high electrical conductivity of the cerebrospinal fluid (CSF) and the irregular shape of the cortex (25–27). In addition to the need of modeling the complex geometry, one of the uncertainties is the fact that human head tissue conductivities (in particular, the most resistive one, the skull) are not well known. They can be estimated with the method of bounded Electrical Impedance Tomography (bEIT) if the geometries are extracted accurately from the structural MRI (28–33).

In general, there are two major electrode types used in TES: large patches for anode and cathode (typically, 5 by 7 cm) and much smaller EEG-like multiple electrodes. In the first more traditional approach, a low number (usually two) of the relatively large round or square patches is used in a bipolar configuration and in different montages (34–36). Large patches help to reduce the current density on the scalp. However, they do not allow reconfiguration of the spatial montages and therefore the stimulation patterns during a stimulation protocol on the brain dynamics time scale (milliseconds). With the advances in dense array EEG, large electrode arrays are now available for non-invasive neuromodulation. The use of a more flexible multi-electrode head harness allows implementation of much more versatile, and even closed loop, dynamical stimulation protocols targeting multiple ROIs at a time, concurrent recording of EEG for neurofeedback, and rapid adjustment of injection configuration patterns in software. With a dense array of electrodes, TES also increases focality and intensity on the cortical targets (37, 38).

By changing two patch electrode positions (or the smaller electrode cluster approximating the patch) on the scalp with the fixed current injection level, one can optimize the current delivery to the cortical ROI using the evolution strategy algorithm (39). We will show in this paper that this optimization problem for directional current density and without imposing the additional constraints of minimal exposure to other brain areas can be solved directly and accurately with the use of the reciprocity principle. Dense array TES optimization is generally more complex due to the much larger number of degrees of freedom than in two patch electrode tDCS, and a requirement to minimize exposure to other than target brain regions. The key challenge is to find a current injection pattern, describing the electrical source or sink current levels for each electrode in a dense electrode array (say, in a 128 or 256 montage) that improves the targeting goal to maximize the current density at the cortical ROI and minimize it at other brain areas. This trade-off problem is mathematically modeled as an optimization problem and solved by means of numerical optimization algorithms such as Least Squares (LS), Linearly Constrained Minimum Variance (LCMV) (37, 40, 41), interior point in disciplined convex programing (37, 42), or genetic algorithms (43). In order to comply with safety constraints, the limiting of the current per electrode requires ℓ_1_-norm type constraints making these algorithms iterative and thus, computationally intensive. Moreover, the solutions in these algorithms require an independent current source for each electrode, increasing the complexity and cost of the associated equipment [except for a low number of studies on dense array tDCS with fewer current sources and fewer number of injecting electrodes (44)].

The reciprocity principle relates the complementarity of the electric field at a cortical dipole location created by injecting a current on the scalp, with the electric potential at the scalp injecting points caused by the same dipole (45–47). It couples the electroencephalography (EEG) and TES forward problems (FP) into an efficient computational solution for EEG source analysis and TES. A similar reciprocity coupling exists between the magnetoencephalography (MEG) and TMS FP as well (48–51). This principle can be used as a guideline for finding current injection patterns in both dense array TES with hardware and safety constraints (52) and in dynamically reconfigurable multicoil TMS (53–55).

With the denser coverage of the scalp in dense array EEG, the poles of EEG topography for any lead field from the cortex are approximated better, and therefore, the reciprocal current injection from those “pole” electrodes are expected to provide a more accurate targeting. The goal of this work is to address the questions of whether the reciprocity-based methods of targeting perform similarly or better than the LS and LCMV methods, and whether the use of 256 electrodes instead of 128 indeed improves the performance of the methods. We extend our preliminary results (52) on the use of the reciprocity principle for obtaining convenient current injection protocols using 128 and 256 high density sensor array EEG nets. We show that the reciprocity method is optimal in maximizing the component of the current density at the target along the desired orientation. We propose and describe four methods derived from reciprocity, taking into account empirically the additional requirements of minimizing TES exposure of non-target brain areas, and contrast them against the LS and LCMV algorithms. We perform simulations in a detailed FE head model considering four representative cortical targets to evaluate the performance of the methods in terms of a targeting error (TE), focality, directionality, and intensity. The first reciprocity based method is of theoretical significance where only one electrode injects the total maximum current and the remaining electrodes act as multiple sinks to spread return currents and minimize TES exposure to non-target areas. The other three reciprocity methods consider an additional restriction: an upper bound in the current delivered by each electrode, which is usually considered as a safety constraint to avoid skin irritation. These methods differ in the way of selecting the sinks and result in better optimization in terms of either the delivered total intensity to a target (the “opposite” configuration) or the focality metric (the “ring” configuration). As it is shown later in the Section “Results,” the global TE of these two methods is dependent on the orientation of the cortical source patch. Given this limitation, we developed another simple algorithm, also based on the reciprocity principle, which is robust against targeting orientations, termed “Reciprocity Opposite Averaged Distance Sink Selection (ROADSS).”

## Materials and Methods

### Head Model

#### MRI and CT Data Collection and Segmentation

A reference model of soft tissues for an adult subject was derived from T1-weighted MR images of the head of a 36-year-old healthy Asian male. It was obtained with a 3T Allegra scanner (Siemens Healthcare, Erlangen, Germany). The bone structure was derived from CT scans of the same subject recorded with a GE CT scanner (General Electrics, Fairfield, United States). The acquisition matrix was 256 × 256 × 256 with a voxel size of 1 mm × 1 mm × 1 mm in both the CT and T1 scans. To build the anatomically accurate head model geometry, the T1 MRI images were automatically segmented into seven tissue types (brain gray matter, brain white matter (WM), CSF, scalp, eyeballs, internal air, and skull). The CT volume was segmented into soft tissues, internal air components, and skull bones, and coregistered to the MRI using the segmentation and image processing package BrainK [Electrical Geodesics, Inc. (EGI)] (20). The typical electrode positions in the 128 and 256 high-density EGI sensor nets montages determined for this subject in previous studies (56) using the Geodesic Protogrammetry System (GPS) (57) were coregistered to the head volume using EGI’s image software BrainK as well.

#### Finite Element Geometry

**Figure 1:**
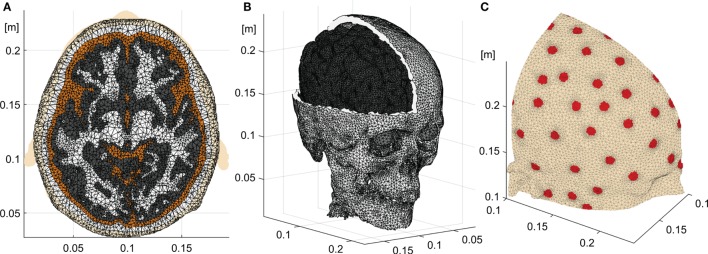
**Head model**. **(A)** Axial slice showing the different tissues of the segmented head and the tetrahedral mesh. **(B)** Skull, eye balls, and gray matter meshes. **(C)** Electrodes on the scalp with the denser mesh on the electrode to skin contact surfaces.

**Figure 2:**
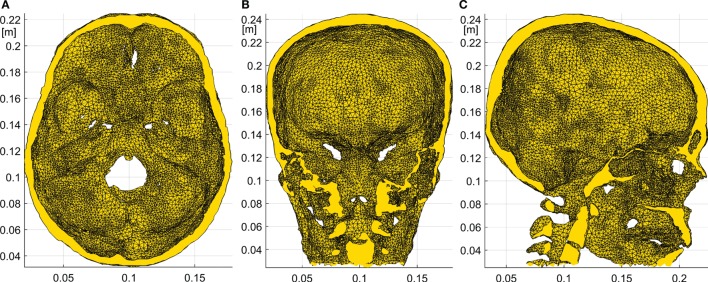
**Details of the skull FE model**. Axial **(A)**, coronal **(B)**, and sagittal **(C)** slices of the skull obtained from the subject specific CT. The skull base and optic foramina as well as the frontal sinuses and other internal air compartments are clearly seen.

A FE tetrahedral mesh of about two million elements was built from the volumetric segmentation using the *iso2mesh* package (58). This moderately large number of elements resulted in a highly detailed head tissue geometry being computationally tractable at the same time. The elements corresponding to the internal air pockets were removed. The skull mesh was obtained directly from the CT segmentation, thus preserving the geometry details of the base of the skull and facial bones including numerous skull foramina and optic canal openings. Special care was taken in smoothing the gray matter and cortical surface extraction to avoid numerical errors when computing the normal cortical vectors. In this study, we used the Complete Electrode Model (CEM) for the electrodes, and the mesh was refined near the electrode contact surfaces. The CEM takes explicitly into account the finite electrode area, top electrode metal cover, and the metal electrode to skin contact impedance through additional boundary conditions (59–61). The typical EGI electrode of 8 mm diameter has a contact resistance of approximately 40 kΩ (62, 63) assuming that the scalp is not scratched. This translates into a contact impedance (*z_l_*) per unit area of about 2.01 Ω × m^2^. The tetrahedral mesh, the segmentation, and the electrode surfaces for the 128 EGI sensor net are shown in Figure 1. Three slices of the skull mesh demonstrating the details of its geometry are shown in Figure 2.

To estimate the quality of the FE mesh, we used the stretch factor metric for a tetrahedral element. The stretch factor is the normalized ratio of the inscribed sphere radius to the maximal edge length, and it is a good measure of how much an element is distorted from an ideal regular tetrahedron. A large number of elements with a stretch factor lower than 0.05 in a 3D mesh would introduce significant numerical errors (64). We have computed this metric for our mesh and found that only about 0.1% of all elements had the stretch factor lower than 0.05. The minimum volume of the elements was also controlled to avoid numerical errors in the potential gradient computation.

#### Tissue Electrical Conductivities

Although our model allows to use anisotropic and inhomogeneous parameters, in this study we assigned isotropic and homogeneous average conductivity values to each separate tissue: 0.2 S/m for the WM, 0.33 S/m for the gray matter (GM), 1.79 S/m for the CSF, 1.5 S/m for the eye balls, 0.008 S/m for the skull, and 0.35 S/m for the scalp. These conductivity values for the head tissues are based on the typical literature data (65, 66).

### Forward Problem

The volume conduction in the low frequency range of EEG and TES is governed by the quasi-static approximation of the Maxwell equations, the Poisson equation (46, 67). Mathematically, the FP in TES assuming the CEM can be stated as follows (59):

(1) $$\left\{\begin{array}{cc} \vec{\nabla }\;\left(\sigma \vec{\nabla }\psi \right)=0 & \text{in}\;\Omega \\ \sigma \;\left(\vec{\nabla }\psi \right)\cdot \hat{n}=0 & \text{in}\;\delta \Omega \\ \psi +z_{l}\sigma \;\left(\vec{\nabla }\psi \right)=V_{l} & \text{in}\;{\text{E}}_{\text{l}}\\ \underset{E_{l}}{\overset{\;}{\int }}\;\sigma \;\left(\vec{\nabla }\psi \right)\cdot \hat{n}\;\mathrm{dS}=I_{l} & \text{in}\;{\text{E}}_{\text{l}} \end{array}\right.,$$

where ψ is the electric potential, σ is the conductivity tensor, Ω is the whole head solid, δΩ is its boundary, which is not in contact with the electrodes, E_1_ is the scalp to electrode “l” contact area, *z_l_* is the contact impedance, $\hat{n}$ is the normal to the boundary vector, *V_l_* is the measured potential at each electrode, and *I_l_* is the injected current. It is worth noting that Eq. 1 is applicable both in the DC and AC mode. At the EEG frequencies (<100 Hz), one can neglect the capacitance effects, so all parameters and variables in the equation system of Eq. 1 (including the contact impedances) are real valued. In the AC mode, the voltages and currents are considered in the sense of AC amplitudes. We solved the system (Eq. 1) numerically, using the first order Finite Element Method (FEM) with the Galerkin approach (68). The problem is reduced to a linear system of equations:

(2) Kv=f,

where **K** is the (*N* + *L*) × (*N* + *L*) “stiffness” matrix (*N* is the number of mesh nodes and *L* is the number of electrodes), **v** is the unknown (*N* + *L*) × 1 vector of the electric potential at each node of the mesh and at each electrode, and **f** is a (*N* + *L*) × 1 vector with the current injection information. The matrix **K** is built considering the geometry, the conductivities of the tissues, and the contact impedance of the electrodes. Detailed information about the **K** and **f** formulations can be found in the context of the EIT FP (30, 59), which is also defined by the Poisson equation and the same boundary conditions of TES. We used a preconditioned conjugated gradient (PCG) algorithm in MATLAB to solve the resulting linear system **Kv = f** (69) with the LU factorization as a preconditioner. Typical solutions required approximately 1,000 PCG iterations (50 s) to convergence with a tolerance lower than 1 × 10^−11^ for the residuals. After having obtained the discretized electrical potential vector **v**, the computation of the electrical field $\left(\vec{E}=-\vec{\nabla }\psi \right)$ and the current density $\vec{J}$ at each tetrahedral element $\left(\vec{J}=\sigma \vec{E}\right)$ is straightforward.

Due to the linearity of the forward volume conduction problem with respect to injected currents, any current injection pattern on the scalp can be constructed as a linear combination of a complete set of independent current injection patterns. If *L* is the number of electrodes, the set must have at least *L* − 1 independent patterns to form a complete set. In the complete set used in this work, each pattern of the set was modeled as an *L*-dimentional vector **p***_i_* with element *i* equal to *I*_max_, (acting as the current source) and the remaining *L* − 1 elements equal to *−I*_max_/(*L* − 1) (current sinks). *I*_max_ is the maximum allowed current. Other sets [see, e.g., Ref. (37)] are possible as well, and lead to the same results. The electric field was computed for every element of the head volume tessellation using each current injection elementary pattern **p***_i_*, resulting in a 3*T* × (*L* − 1) transfer matrix **T**, where *T* is the total number of elements or tetrahedrons in the head model. The whole TES FP is solved, when the complete set of forward solutions is computed (generally, *L* − 1 independent solutions for an *L* electrode montage).

### Inverse Algorithms

The inverse problem in TES can be stated as follows: given a subject head model, including dense array electrode positions, a ROI on the cortex or in subcortical structures to be stimulated, a spatial profile of the desired impressed electrical field ${\vec{E}}_{\text{t}}$ on the target and ${\vec{E}}_{\text{nt}}$ in the rest of the head, find the optimal current injection pattern on the scalp producing the best approximation of the desired directional current density $\vec{J}=\sigma {\vec{E}}_{t}$ on the brain target. If ${\vec{E}}_{\text{t}}$ is given in the way that it is maximal on the target in the desired direction and minimal elsewhere $\left(\left|{\vec{E}}_{\text{t}}|\gg |{\vec{E}}_{\text{nt}}\right|\right)$, the problem can be reformulated also simply as: maximize the impressed directional density on the target and at the same time, minimize it in other directions on the target and elsewhere in the brain and in the head. Finally, one needs to comply with safety constraints (limiting the total injected current into the head and the current per each electrode), and with hardware constraints (the number of independent sources).

#### Parallels of TES Inverse Problem with Distributed Source Localization in EEG

The inverse problem in TES has similarities with the inverse problem in distributed source localization in EEG (70, 71). Indeed, *L* sensor potentials on scalp in EEG, **y**, are produced due to the weighted sum of *N* equivalent cortical dipoles (the cortex is assumed to be parcellated into small subdomains or patches): **y** = **Ms** + noise, where **s** is a vector of dipole current densities, and **M** is an *L* × *N* lead field matrix describing potentials on the scalp produced by a unit dipole strength. Similarly, the impressed electric field spatial profile **e** desired on the *T* tetrahedrons inside the whole head volume due to injection current into *L* sensors on scalp in TES is governed by a linear relationship: **e** = **Tc** + noise, where **T** is the 3*T* × (*L* − 1) aforementioned transfer matrix created by each individual **p***_i_*, and **c** is a vector of individual coefficients *c_i_* such that the sum $\sum_{i=1}^{L-1}$ **p**_*i*_*c*_*i*_ is the vector of individual injection currents in each electrode that generates **e**.

In both cases, the inverse solutions can be formally stated analytically in a similar manner as:

(3) $$s_{\text{opt}}=\underset{s}{\text{argmin}}\;∥Ms-y{∥}^{2}=M^{\text{T}}{\left(MM^{\text{T}}\right)}^{-1}y,$$

for the EEG case and

(4) $$c_{\text{opt}}=\underset{c}{\text{argmin}}\;∥\mathrm{Tc}-e{∥}^{2}={\left(T^{\text{T}}T\right)}^{-1}T^{\text{T}}e.$$

for TES. The crucial difference is that in EEG source localization (Eq. 3), we have the underdetermined case (number of unknowns *N* ≫ *L*, the number of equations) of the minimum norm solution, which is generally ill-posed, vulnerable to noise, and therefore requires some form of regularization (71). In contrast, for the inverse problem in TES (Eq. 4), we have the overdetermined case [number of unknowns (*L* − 1) ≪ 3*T*, the number of equations] where the exact LS solution is well posed.

#### Least Squares

Assume a target comprising elements *t*_1_, …, *t_R_* (*R* ≪ *T*) and a three element vector **d** as the desired targeting orientation. We form a target selection vector **b** of *T* × 1 with “ones” in the *t*_1_, …, *t_R_* elements and “zeroes” in the rest. Then, we form the objective electric field vector **e** of 3*T* × 1 as: **e** = **b** ⊗ **d**, where ⊗ represents the *Kronecher product*, i.e., vector **d** is replicated in **e** for each target element. This imposes the desired orientation at each element of the target. The LS solution **c**_LS_ is obtained using Eq. 4. The LS optimal pattern **p**_LS_ is the linear combination of the **p***_i_* patterns weighted by the elements of **c**_LS_, i.e., ${\text{p}}_{\text{LS}}=\sum_{i=1}^{L-1}\;c_{i}{\text{p}}_{i}$. Finally, **p**_LS_ is scaled such that the total injected current is fixed to *I*_max_, i.e., ${\text{p}}_{\text{LS}}=2I_{\text{max}}\sum_{i=1}^{L-1}\;c_{i}{\text{p}}_{i}∕{∥\sum_{i=1}^{L-1}\;c_{i}{\text{p}}_{i}∥}_{1}$, where ∥⋅∥_1_ is the ℓ_1_ norm.

#### Linearly Constrained Minimum Variance

The LCMV approach enforces a hard constraint, the desired electric field at a particular spatial point **r**, and it minimizes the overall power of the electric field in the rest of the head. The mathematical formulation is:

(5) $$c_{\text{LCMV}}=\underset{c}{\text{argmin}}\;∥Tc{∥}^{2}\;\text{ subject to}\;\tilde{T}c=d,$$

with solution:

(6) $$c_{\text{LCMV}}={\left(T^{\text{T}}T\right)}^{-1}{\tilde{T}}^{\text{T}}{\left(\tilde{T}{\left(T^{\text{T}}T\right)}^{-1}{\tilde{T}}^{\text{T}}\right)}^{-1}d,$$

where $\tilde{T}$ are the three rows of **T** corresponding to the element at position **r**. The optimal pattern for the LCMV method **p**_LCMV_ is also derived as the linear combination of **p***_i_* weighted by **c**_LCMV_ and scaled to consider the total current limit.

The LS and LCMV solutions described here are the simpler versions of the LS and LCMV formulations described in Ref. (37). They do not consider an upper bound for the current at each electrode. Thus, these simpler versions do not need iterative solvers and are mainly used in this work as a ground truth for the reciprocity-based methods that do comply with the current limit per electrode safety constraint.

#### Reciprocity

The reciprocity theorem coupling TES and EEG (46, 47) states that given a dipole at position **r** with dipolar moment **d**, the electric potential (Φ) difference between any points *a* and *b* on the scalp can be computed as the dot product:

(7) $$\Phi \left(a\right)-\Phi \left(b\right)=\frac{d\cdot \vec{\nabla }\psi_{\mathrm{ab}}\left(r\right)}{I_{\mathrm{ab}}}$$

where ψ*_ab_*(**r**) is the resulting potential at location **r** when an electric current I*_ab_* is injected at the arbitrary points *a* and *b*. It can be immediately deducted, that if points *A* and *B* are the poles of the forward scalp potential topography produced by dipole **d**, Φ(*A*) – Φ(*B*) reaches the maximum, therefore, the dot product of **d** and impressed potential gradient at **r** are also maximal. This means that injection of the given current amplitude *I_AB_* into the EEG topography poles maximizes the directional (along dipole **d**) current density $\left(\vec{J}\;=\;-\sigma \;\vec{\nabla }\psi_{\text{AB}}\right)$ on the target location **r**. Mathematically,

(8) $$\begin{array}{ll} A,B & =\underset{a,b}{\text{argmax}}\;\left\{\Phi \left(a\right)-\Phi \left(b\right)\right\}=\underset{a,b}{\text{argmax}}\;\left\{\frac{\vec{\nabla }\psi_{\mathrm{ab}}\left(r\right)}{I_{\mathrm{ab}}}\cdot d\right\}\Leftrightarrow \vec{\nabla }\psi_{\mathrm{AB}}\left(r\right)\cdot d\text{ is maximal}. \end{array}$$

**Figure 3:**
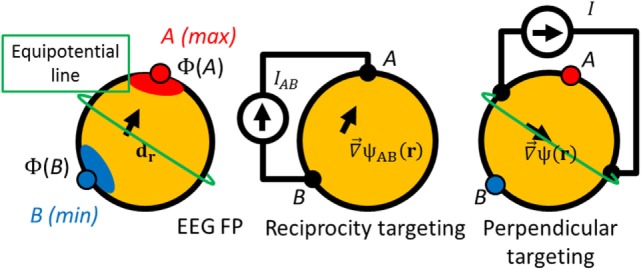
**Schematics of the reciprocity targeting**. Left: a simulated dipole at position *r* with moment d_r_ generates a potential distribution Φ with a maximum (point A), a minimum (point B), and a isopotential lines (like the green one). Center: poles A and B can be used for the current injection maximizing the component of the gradient of the potential $\vec{\nabla }\psi_{\text{AB}}$ in the direction **d_r_**. Right: if any injection pair is on an isopotential line [Φ(*A*) − Φ(*B*) = 0], the current density on target is perpendicular to the target orientation.

It is also true that injection into any points *A* and *B* belonging to the EEG topography isopotential lines [Φ(*A*) – Φ(*B*) = 0] minimizes the collinearity with the impressed current density [in accordance with Eq. 7, the dot product on the right side is equal to 0, meaning that the impressed current density along **d** is also 0]. It is important to emphasize that the reciprocity principle does not provide immediate information about the total impressed current density. In the reciprocity principle, the dense array EEG lead fields for a given unitary cortical dipole can serve as a guidance for non-invasive TES of the cortical patch represented by that dipole: the dot product $\left(d\cdot \vec{\nabla }\psi_{\text{AB}}\right)$ and therefore the normal component of the current density on the cortex is maximized when current is injected into the top of scalp EEG topography referenced to *B*, and vice versa, the normal component is minimized when the current is injected into the points belonging to the same isopotential line of the scalp EEG topography, as explained in Figure 3.

Thus, (*A, B*) is the pair of points on the scalp that maximize the component of the gradient of the potential ψ along the desired orientation **d** at **r**, and ideally these points should be used in the neurostimulation based on the reciprocity principle. With a larger number of electrodes there is a better coverage of the scalp, and so, a better approximation for the poles of EEG topography. Consequently, we expect better targeting accuracy based on the reciprocity method with more electrodes.

To comply with the limits for current levels applied per one EEG electrode, one can distribute the injected current over several electrodes surrounding the optimal reciprocity points, *A* and *B*. Moreover, TES targeting generally requires not only large directional intensity on the target, but also focality to comply with the minimal exposure of non-target brain areas. To take this requirement into account empirically, one can distribute return current electrodes (sinks) for preferential anodal targeting in tDCS over as many electrodes as possible.

It is worth noting that the reciprocity principle has been utilized in the modern EEG forward solvers (72–74) to accelerate the calculations of the lead fields needed in the distributed inverse EEG problem; however, it has not been used in the context of tDCS targeting. Only recently, Salman et al. (74) developed efficient methods for computing lead field matrices for EEG, transfer matrices for TES, and auxiliary matrices for bEIT all in one unified approach. This approach draws on the reciprocity principle for a given head model, such that additional computation for these multiple measures require only additional input/output operations. Using the reciprocity principle (Eq. 7), the lead fields from *N* dipoles to *L* sensors can be computed instead of sequential forward solutions for each dipole (totaling to *N*), by injecting unit currents into *L* − 1 electrode pairs and computing impressed potential gradients at each dipole location followed by a fast vector scalar multiplication for each dipole to calculate a potential at a given electrode. With this efficiency, the computation is about 100 times faster (typically number of electrodes *L* ~ *N*/100).

Below, we present four methods based on the reciprocity principle to determine optimal current injection patterns in TES. Except for the first one, called “one source,” the other three consider the safety restriction that the current intensity at each electrode does not exceed *I*_max_/10, i.e., the total current injection in TES (typically <2 mA) is spread over at least 10 EEG electrodes with the typical contact surface area of about 50 mm squared. This spreading reduces current densities on the scalp immediately under electrodes and therefore the current perception threshold in subjects. Because we want to inject the current following as much as possible reciprocity (point A), and reducing the hardware requirements, the number of electrodes is reduced to the minimum (10 in our case) and they are clustered, i.e., the 10 electrodes with the highest EEG potentials are selected as sources. This constraint and the upper limit of the current at each electrode imply that all the sources deliver the same amount of current. The number of sinks might be larger than 10 to avoid high current densities near the sink region. This intended spreading implies that the current is evenly distributed also in the sinks. Note that this can be done with independent sources but also it would be possible to use only one physical source connected to all sinks and sources if assuming equal contact impedances. Because the contact impedance is usually much higher than head resistivity, they will dominate the current distribution, and it will balance according to the contact impedance of each channel.

Specifically, we have considered the following cases:

(1) *One source configuration*: for a target at position **r** with desired orientation **d**, outward to the cortex surface, we simulate a dipole at **r** with a unit magnitude and collinear to **d**, and solve the EEG FP. The EEG FP is solved numerically using the same mesh and also the first order FEM with the Galerkin approach. The equation system and boundary conditions are similar to Eq. 1 but with the electromagnetic source (dipole in this case) inside the volume and not on its boundary. To model the dipolar source in FEM, the volume integral equation for the tetrahedron containing the dipole is solved, modifying the elements of the vector **f** (Eq. 2) corresponding to the four nodes of that specific tetrahedron (75). The electrode with the maximum electric potential is chosen as the current source and all other electrodes (127 or 255) are selected as sinks. This configuration aims at increasing the current density at the target by injecting the maximum current in the best electrode according to reciprocity but reducing the current density in the rest of the head spreading the sinks as much as possible. It does not comply with the current density limit per electrode safety constrain, but it is interesting to compare with the cases of LS and LCMV optimization.
(2) *“Opposite” configuration*: the EEG FP is solved as in the previous case, but now the 10 electrodes with maximum electrical potential are selected as sources, and the 30 electrodes with minimum electrical potential are selected as sinks. We choose 30 electrodes instead of 10 to have some spreading of the current density near the sink zone. In this configuration, the maximum current per electrode is *I*_max_/10 (typically <200 μA per electrode) as allowed by the safety restriction. In this configuration, the sinks are selected also accordingly to the reciprocity principle (near point B), but the number of sinks is increased to spread more the current density near the sink region than near the source region.
(3) *“Ring” configuration*: the previous method is appropriate to increase directionality but, as it will be shown later, it is not optimal in reduction of the current density in the rest of the head. In order to increase focality, the “ring” version of the reciprocity method is analyzed. The 10 sources are selected exactly as in “*opposite”* configuration, but sinks are chosen as the 10 closest electrodes forming a ring around the sources. This is similar to the “ring” 1 source to 4 sinks configuration analyzed in Ref. (37). In this configuration, the sinks are not selected according to reciprocity, but all the current is concentrated near the region to stimulate, and almost no current is delivered to the rest of the head.
(4) *“ROADSS”*: as it will be discussed later, the Targeting Error (TE) of the “*opposite*” and “*ring*” configurations is dependent on the target orientation. We have developed a new version of the reciprocity-based targeting methods that combines both configurations such that it performs similar to the “*opposite”* method when the target is parallel (tangential) to the scalp and similar to the “*ring”* method when the target is perpendicular (radial) to the scalp surface. In this approach, the sources are selected according to reciprocity in the same way that in “opposite” or “ring” configurations. The distance $d_{r_{\text{m}}r}$ between the centroid of the clustered sources **r**_m_ and the target position **r** is computed. It is expected that this distance is large for tangential targets and small for radial targets. Then, an opposite point **r**_s_ is obtained at the same distance from the target but in the opposite direction following the targeting orientation. If $d_{r_{\text{m}}r}$ is large (tangential), **r**_s_ will be far from the target in the opposite direction, so the closest electrodes to **r**_s_ are selected as sinks thus leading to a configuration similar to the “opposite” configuration. On the contrary, if $d_{r_{\text{m}}r}$ is small (radial target), **r**_s_ will be near the target, but the closest electrodes to **r**_s_ are already sources. Then, the sinks are selected as the closest electrodes to **r**_s_ not being already sources. This leads to a “ring” like configuration.

In summary, the *ROADSS* algorithm can be implemented as follows:

- Pick the 10 sources as in the “*opposite”* or the “*ring”* configurations.
- Compute the position **r**_m_, as the mean of the positions of the ten electrode sources.
- Compute the distance $d_{r_{\text{m}}r}$ between **r**_m_ and **r**.
- Compute a new point **r**_s_ as $r_{\text{s}}=r-d_{r_{\text{m}}r}d$ (**d** is unitary and oriented outward from the cortex).
- Pick the sinks as the ten electrodes closer to **r**_s_, that are not already sources.

**Figure 4:**
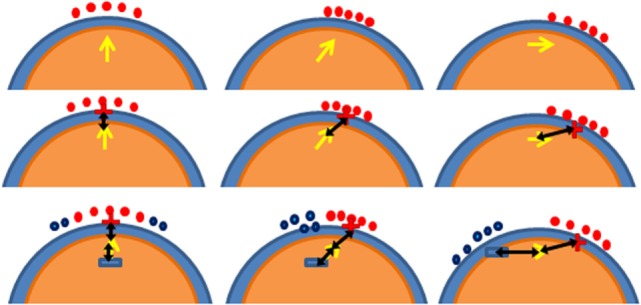
**Schematics of the ROADSS approach**. The three columns represent three different targets: radial (perpendicular), oblique, and tangential to cortex orientations at position **r**. The first row shows the selected sources based on reciprocity (reed dots); the second row shows **r**_m_ (the red “+” sign) and $d_{r_{\text{m}}r}$ as a black arrow; and the third row shows **r**_s_ (blue “–” sign) and the sinks in each case (blue dots). It is clearly seen that in the radial case (first column), the resulting pattern is similar to the “ring” approach and that in the tangential case (third column), the resulting pattern is similar to the “opposite” approach.

The rationale of this method is schematically explained in Figure 4.

Note that the reciprocity methods do not require the computation of the full matrix **T**, and do not require the full matrix inversion as the LS and LCMV methods do. Instead, the reciprocity targeting makes a use of the lead field EEG for an oriented dipole at the target. These lead fields can even be precomputed for the all possible oriented dipoles on the cortex parcellated into patches. The EEG lead fields are also often precomputed for “triples” [dipoles components along *x*, *y*, *z* on the regular grid in the brain GM (74)]. A linear combination of the weighted triples lead fields can be constructed to reproduce a lead field for any desired dipole direction and can be used for effective targeting. If it is required to target ROIs in the subcortical regions of the brain where EEG lead fields are not yet precomputed, it is still possible to use the reciprocity principle by formally placing dipoles in those ROIs, calculating the forward solutions on the scalp and selecting the pole of those solutions for current injecting. Thus, the reciprocity based approach for neurostimulation presents a significant reduction of computational cost and memory. Obviously, in direct current neuromodulation, sources and sinks may be reversed to reverse the current flow.

### Performance Metrics

In order to compare the performance of the different methods, we defined metrics of performance to account for intensity, TE, directionality, and focality. The intensity at the target can be directly obtained as the current density at the ROI of the FP solution. The normalized dot product between this current density and the desired orientation gives the directionality. We also use the projection of the current density along the desired orientation as a metric of intensity and directionality.

The TE was defined as the distance between the geometrical center of the actual ROI and the center of gravity in the imprinted total current densities on the cortex. We computed the global centers of gravity (CoG) as the weighted average of the central positions of the GM elements with an absolute imprinted current density larger than a threshold value of 75% of the absolute maximum (76). It is noted, that while this metric describes a relation between the total current density delivered to the brain and the location of a target, it might not agree well with the targeting ROI by the local maxima in the cortical current density due to the fact that current densities may increase near clustered sinks at the opposite side of the brain far away from a target. Therefore, we computed also the “local CoG” in the same way, but only considering the immediate neighborhood of a target (elements within 3 cm to the true target position) and obtained the TE between the target central position and the “local CoG” in that neighborhood.

The term “focality” can be used to quantify the amount of current density outside the region of interest and, at the same time, the precision (or concentration around the maximum). There is not a unique way of defining a metric for focality. The most intuitive way to compare concentration is to assume an upper threshold in the current density maps on cortex. Then, only one concentrated spot means good focality, whereas big islands or several isolated islands give an idea of more poor focality. Another possibility is to compute the radius of a sphere, centered at the target that contains half of the total current density in the GM (37). This metric is similar to the half-width at half maximum metric used in physics and it is mostly dominated by the current density in the rest of the head (the “tails” of the current density distribution). We will also refer to it as the “global” focality metric. Note that this metric is not centered at the maximum of the current density but on the true target, so it is related to the TE. The dispersion of targeting is usually quantified independently from the bias (or TE in this case). So, we defined another metric to quantify the local focality, F_LOC_, as the ratio between the total amount of current density in a 1 cm radius sphere centered at the “local CoG” and the current density in a 3 cm radius sphere centered at the target. The idea with this metric is to quantify the sharpness of the local current density maximum closest to the true target. Mathematically,

(9) $${\text{F}}_{\text{LOC}}=\frac{\sum_{i\in ϒ\;\;}{\left|\sigma \nabla \psi \right|}_{i}V_{i}}{\sum_{i\in \Gamma }{\left|\sigma \nabla \psi \right|}_{i}V_{i}},$$

where Y contains the GM elements in the 1 cm radius sphere, Γ contains the GM elements in the 3 cm radius sphere, |σ∇ψ|*_i_* is the current density of element *i*, and *V_i_* is the volume of element *i*.

## Results

**Figure 5:**
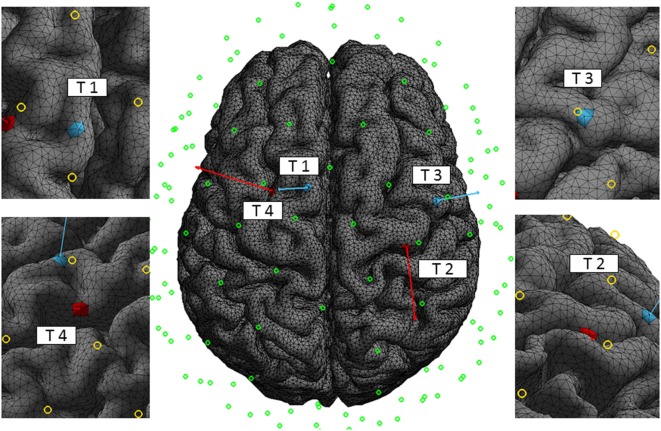
**Targets**. The four trial targets on the cortex, T1, T2, T3, and T4, are shown with the corresponding normal vectors of 5 cm length for better visualization. Normal vectors of targets T1 and T3 are mostly perpendicular and normal vectors of targets T2 and T4 are mostly tangential to the scalp surface. The circles indicate the central position of the electrodes of the 128 EGI sensor net. The normal vector projections to the scalp for T2 and T3 are close to a specific electrode in the 128 montage, while the similar projections for T1 and T4 are in between. The left and right figures are close-up views of the targets with their corresponding normal vectors.

Four trial cortical targets (Figure 5) have been selected to test the reciprocity-based targeting methods and compare them with the reference LS and LCMV methods: T1 is in the medial premotor cortex, T4 is in the premotor cortex as well, but more laterally, and T2 and T3 are in the primary motor cortex (the hand and face areas). In addition, we compared accuracy and efficacy of targeting for two cases of dense array montages (128 and 256 electrodes) for the same total injected current, *I*_MAX_ = 1 mA. All four targeted ROIs had been chosen to have essentially different locations and cortical patches orientations with respect to the scalp landmarks and electrodes. We chose two targets to be on the crowns of gyri, where the cortex surface is almost parallel to the scalp, such that the targeting orientation is perpendicular to the scalp or radial (T1 and T3), and two targets on the walls of sulci, where the cortex is almost perpendicular to the scalp surface and the surface normal vectors are mostly tangential (T2 and T4). Each ROI comprised from 10 to 15 tetrahedrons in a volume of approximately 20 mm^3^. The normal vector for each cortical ROI was computed as follows: first, for each triangular facet within the cortical ROI, the normal vector was computed as the cross product of two of its edges and then, the result was averaged over all GM–CSF interface facets comprising the target patch surface. In the case of T2 and T3, ROIs were almost directly under one of electrodes in the 128 EGI sensor net, while in the case of T1 and T4, ROIs were in between electrode projections to the cortex. The target patches and their normal outward vectors are shown in Figure 5.

**Figure 6:**
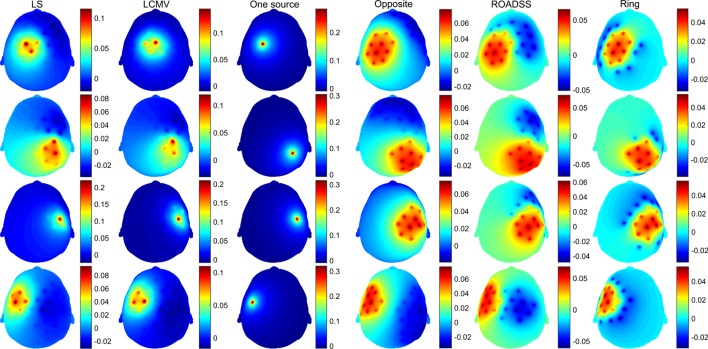
**Imprinted electric potential [V] on the scalp for the 128 EGI electrode net**. Rows from top to bottom correspond to optimal stimulation of targets T1, T2, T3, and T4; columns correspond to the optimal current injection patterns obtained, from left to right, with the LS, LCMV, “one source,” “opposite,” “ROADSS,” and “ring” methods. The total current injected in each case is 1 mA.

**Figure 7:**
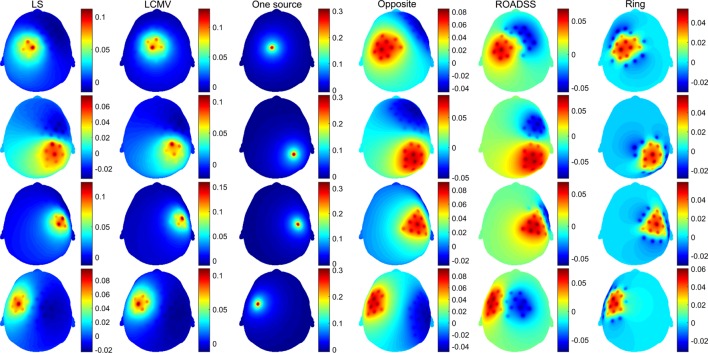
**The same as in Figure 6 for the 256 EGI electrode net**.

**Figure 8:**
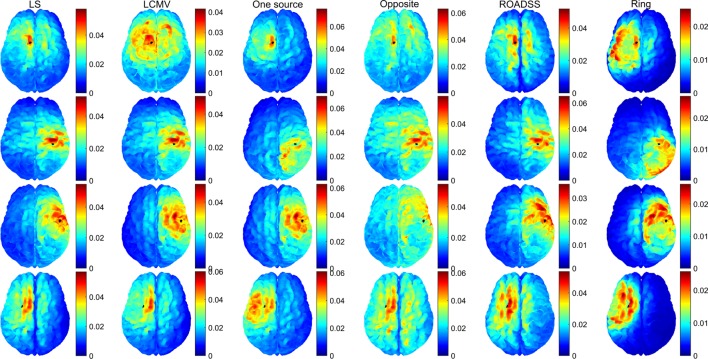
**Module of the total delivered current density on the cortex for the 128 EGI electrode net in ampere/square millimeter**. Rows from top to bottom correspond to optimal stimulation of targets T1, T2, T3, and T4; columns correspond to the optimal current injection patterns obtained, from left to right, with the LS, LCMV, “one source,” “opposite,” “ROADSS,” and “ring” methods. The total current injected in each case is 1 mA. The cortical ROIs to target are shown in black.

**Figure 9:**
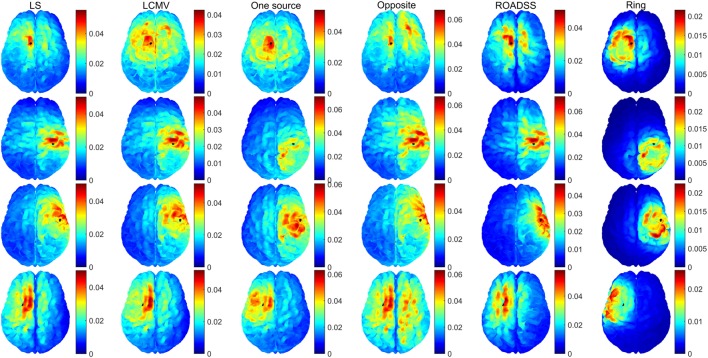
**The same as in Figure 8 for the 256 EGI electrode net**.

In Figures 6 and 7, we show the electric potentials imprinted on the scalp for all optimal current injection patterns found using the 128 and 256 sensor array nets, respectively. The configuration of the optimal pattern for each algorithm is reflected by the relative brightness of the activated electrodes. The exact injected optimal current patterns for each electrode are also shown in Figures S1 and S2 of the Supplementary Material. Figures 8 and 9 show the current density delivered to the cortex for the 128 and 256 sensor nets, respectively. Note that in most of the cases, the global maximum of the current density is close to the target. Also note that the maximum amplitude of the current density on cortex varies significantly with different patterns (the different color scales).

**Figure 10:**
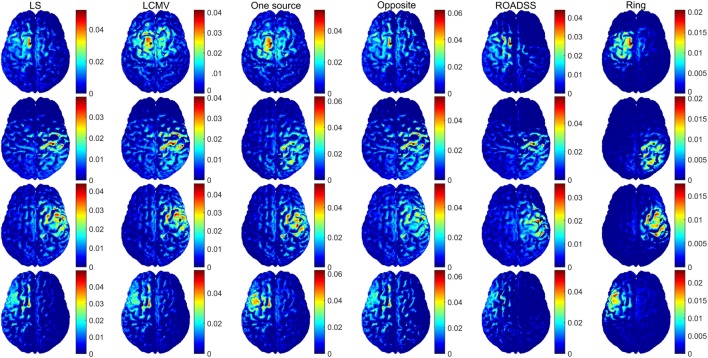
**Normal to cortex component of the current density (ampere/square meter) for the 256 EGI electrode net**. Rows from top to bottom correspond to optimal stimulation of targets T1, T2, T3, and T4; columns correspond to the optimal current injection patterns obtained, from left to right, with the LS, LCMV, “one source,” “opposite,” “ROADSS,” and “ring” methods. The total current injected in each case is 1 mA. The cortical ROIs to target are shown in black.

In Figure 10, we show the normal to the cortex component of the current density for 256 electrodes. Although the physiological effects of the current orientation on the target are still under study, it is believed that the normal to cortex surface component of the current density, following the predominant orientation of the pyramidal cells, is the most important one in TES (19, 77). Note that if only the normal to cortex component of the current density is considered (Figure 10), the maximum of the current density is much closer and substantially more concentrated at the target than in the case of the total current delivered to ROI as shown in Figures 8 and 9.

**Figure 11:**
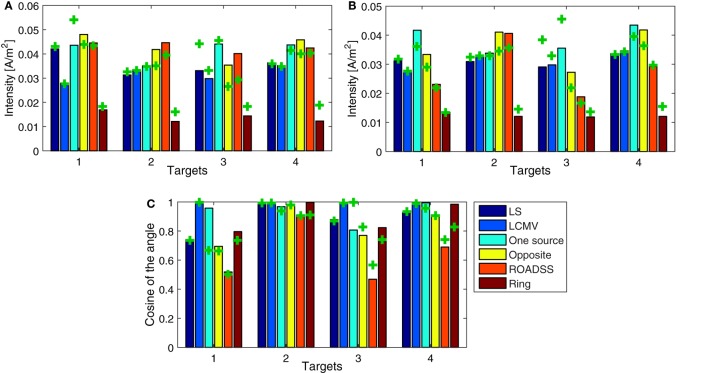
**Comparison of quantitative metrics of targeting between different methods. (A)** Module of the total current density at each target; **(B)** directional current density on the target as the dot product between the current density and the unitary normal vector at each target surface; and **(C)** the normalized dot product between each target normal and the resulting current density. The bars depict the results for the 256 electrode net while the “+” marks correspond to the 128 sensor net. The total current injected in each case is 1 mA.

For each target and each method, we have computed the quantitative metrics based on the total current density delivered to a target and its directionality, TE, and focality. The total current densities on the targets are shown in Figure 11A, and their projections to the cortical normal vectors are shown in Figure 11B. The normal component of the imprinted current density normalized to the total current density is depicted in Figure 11C. Note that better directionality overall implies lower differences between Figures 11A,B or equivalently, a value closer to one in Figure 11C.

**Figure 12:**
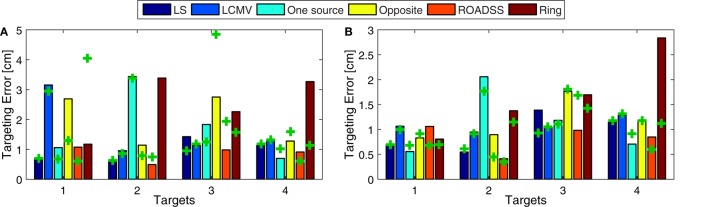
**Targeting Error (TE)**. Distance between the global **(A)**, and local **(B)** center of gravity and the true central position of the target, for the 256 EGI electrode net (bars) and for the 128 EGI electrode net (“+” marks). Global TEs larger than 5 cm were removed for clarity.

The global TE metric is shown in Figure 12A and the “local TE” in Figure 12B. Note that if we consider the normal to cortex component shown in Figure 10 instead of the total current density in Figures 8 and 9, there are fewer maxima distant from the target and the corresponding TE is lower.

**Figure 13:**
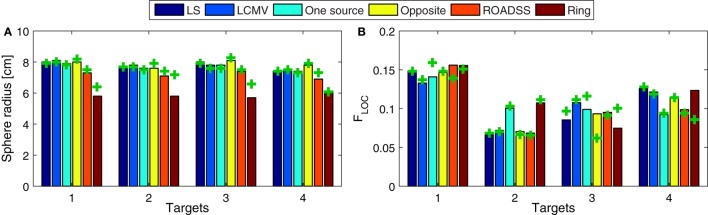
**Focality. (A)** Focality defined with the half-width at half-maximum radius approach for the whole gray matter domain (the lower the better), i.e., a global focality metric; and **(B)**, the local focality (the larger the better) as defined in Eq. 9. The bars depict the results for the 256 electrode net while the “+” marks correspond to the 128 sensor net.

The global focality metric is shown in Figure 13A and the local F_LOC_ metric is shown in Figure 13B, where a larger F_LOC_ indicates better focality. Again, the F_LOC_ metric would highly improve if one can consider only the normal to cortex component of the current density, as can be seen from Figure 10.

In addition, we have simulated the optimal solutions in the LS and LCVM methods with a reduced transfer matrix of **T** representing only cortical elements. The results were unexpected at the first glance: we observed multiple spurious solutions for active electrodes in the facial and neck area. However, the more detailed analysis showed that those solutions always appeared in pairs of sources – sinks which canceled each other, and which did not reach the cortex (and therefore did not impact on the current densities on the cortex). That is, to avoid these spurious solutions in the LS and LCMV approaches, it was needed to minimize the electric field in the non-target elements of the whole head (including the other tissues than GM) and not only in the non-target elements of the GM. In contrast, the reciprocity based methods worked well with the reduced transfer matrix.

One important factor observed in these simulations was the importance of accurate tissue geometry for modeling the influence of CSF on current delivery to the cortex adjacent to CSF (Figure S3 in Supplementary Material). Interestingly, the FE simulation showed that narrow regions of CSF (gray circles in Figure S3 in Supplementary Material) were associated with high current densities, with the effect appearing similar to the Bernoulli principle of higher velocity with restricted flow in hydrodynamics. Such CSF effects are important in cortical regions (black circles in Figure S3 in Supplementary Material) adjacent to high current densities in CSF.

### Impact of the Skull Conductivity on Targeting

We also conducted simulations with variations of the skull conductivity. We found that the delivered current density to the cortex (and therefore the dose) for the same electrode configuration and target is highly dependent on this parameter, as seen in Figure S4 in Supplementary Material. Also, for any skull conductivity value from the computed range, the total current density delivered to the shallow targets T1 and T3 are around 35% larger than the densities delivered to the deeper targets T2 and T4. These results emphasize the importance of the skull conductivity calibration for each individual subject, using, for example, bEIT (29, 30, 33).

## Discussion

### Injection Patterns

Both the LS and LCVM methods produced rather sparse (in terms of the injected current amplitude distributions) optimal solutions for activating the electrodes on the scalp, as it is seen from Figures 6 and 7 and Figures S1 and S2 in Supplementary Material. As it might be expected, these sparse or focal scalp distributions were more pronounced for the ROIs whose cortex orientations were perpendicular to the scalp (T1 and T3). The constraints imposed on the maximum current injected per electrode obviously would make these patterns more spread and the total delivered currents to the targets would be reduced as well. Interestingly, the simple reciprocity based configuration, with only one source placed on the top of the EEG positive topography pole and multiple sinks distributed evenly over the rest of electrodes (column 3 in Figures 6 and 7), produced effects that were very close to the brightest electrodes found in the optimal LS and LCMV solutions. One can conclude that the LS and LCMV optimizations were effective in reproducing the efficient characterization of the source targeting captured by the reciprocity principle, and this is a key finding of this work. The other reciprocity methods, like the opposite one (10 sources and 30 sinks), and the ring methods (10 sources surrounded by 10 sinks) are visualized in columns 4 and 5 of Figures 6 and 7. One can see that grossly they are variations of the first reciprocity method, differing only in the way of distributing the total injected current of 1 mA to comply with the safety requirements (such as less than 100 μA per electrode). It interesting to note, that while the ROADSS method pattern shown in column 5 in Figures 6 and 7 is also distributed in compliance with the safety requirement, it has the largest correlation of the injection pattern with the LS and LCMV solutions, and this is another very important result of this work.

### Current Density

One can observe from Figures 8 and 9, that, in general, the cortex current densities obtained with the reciprocity methods are similar to the densities obtained with the LS and LCMV methods that are not constrained, but assume different and unbounded current injection magnitudes in each electrode. This is especially true with the “one source” and ROADSS methods, the “opposite” method for tangential targets (T2 and T4), and the “ring” method for radial targets (T1 and T3). Also the maxima close to the targets obtained with the LS or LCMV methods usually coincide with a local maximum of the reciprocity methods. This effect is clearly visible in Figure 10, where the distributions of the normal component of the current density are very similar across different methods. Due to the constraint of limiting the current per electrode, the reciprocity methods distribute the injected current into a larger number of electrodes, i.e., a larger area. This necessarily produces more maxima away from the target. However, comparing Figures 8 and 9, it can be seen that the use of more electrodes reduces the number and amplitude of these maxima, except in the specific case of the “opposite” configuration.

### Intensity and Directionality

Figure 11 represents the quantitative metrics of total and directional (normal to the cortex) current density delivered to all four targets using all six optimization algorithms. It is clearly seen that in most cases, the one source, the opposite, and the ROADSS reciprocity-based methods outperform the LS, LCMV, and “ring” reciprocity methods in terms of delivered current densities to the targets. It is important to note that a larger spatial distance between sources and sinks allows more of the injected current to pass through the skull. In contrast, for electrode arrangements with nearly adjacent sources and sinks, most of the current flows through the scalp and little current reaches the brain surface. These factors explain why the ring method has poorer performance on the intensity metrics (Figure 11). With more options for electrode selection in relation to the complex geometry of the folded cortex, the 256 net is, in general, better than the 128 net for current delivery to the cortex. In addition, the greater electrode density allows a higher concentration of sources, even when current is limited to 100 μA per electrode. Note that for the reciprocity methods, the cross markers corresponding to the 128 electrode net are generally below the bars corresponding to the 256 electrode net in Figure 11A,B, except for the “ring” case where in the 256 montage, sources, and sinks are closer thus leading to lower current density reaching the cortex.

The degree of directionality of delivered current densities for different algorithms can be seen in Figure 11C as the percentage of the total current delivered normally to the target patch, as well as in the brightness of the color map of the normal to cortex current densities in Figure 10. One can see that in several cases almost 100% of the delivered current is directional, so the directionality metric is fairly high in the most of algorithms and target cases. As expected, and in agreement with Dmochowski et al. (37), the LCMV solution presents the maximal directionality as it is enforced by the hard constraint of Eq. 3. Note that the “one source” approach also has a very good directionality, consistent with the theoretical analysis of reciprocity given in Section “Reciprocity,” followed by the “opposite” and the “ring” configurations. The ROADSS method directionality is not as good (50% for targets T1 and T3), but even then, the current densities in the desired orientation are in general similar to the LS and LCMV methods (Figure 11B). Finally, the directionality increases to some extent with 256 electrodes. Directionality can be also estimated qualitatively by comparing the imprinted total and directional current density cortical maps in Figures 9 and 10. Because the cortical columns and their pyramidal neurons are perpendicular to the cortical surface, it seems likely that the effective current dose will be the perpendicular component (surface normal) as well, although this assumption must be tested explicitly in dosage studies that relate the behavioral or electrophysiological effects of TES to both the total current density and the surface-normal current density doses.

### Targeting Error

It can be seen in Figures 8, 9, and 12A that the TE is in general low for the LS and LCMV methods, as well as for the hardware and safety constrained reciprocity based methods, except for some cases where the absolute global maximum is somewhat far from the target. This appears to be caused by deep “hot spots” due to complex cortical geometry and the high conductivity of CSF (see for example the high current density spot on the right frontal lobe in T1 LCMV 128 net case in Figure 8, top row, second column). The similar inaccuracies appear to be caused by larger current density near the sink electrodes in the “opposite” reciprocity configurations. In the case of radial to scalp targets (T1 and T3), the “opposite” configuration is not optimal but the “ring” configuration has a lower global TE. Conversely, the “opposite” configuration performs better in terms of the global TE than “ring” configuration for tangentially oriented targets (T2 and T4). This is also expected: in tangential targets, the poles of the EEG topography on the scalp lie far from the target, and the “ring” configuration concentrates the current density in a region close to the targets and sinks.

In contrast, the ROADSS configuration, in accordance with its design, has a lower global TE regardless the target orientation, and this is a very important result of this work as it was the major motivation for developing the ROADSS method. Note that in Figures 6 and 7, the ROADSS current injection patterns are more similar to the “opposite” patterns for the tangentially oriented targets (T2 and T4) and more similar to the “ring” configuration for the radially oriented targets (T1 and T3). In Figure 12B, it can be observed that the “local TE” is in general lower than 1 cm and that in all cases, there are at least two reciprocity configurations that performs similarly to the more flexible LS and LCMV methods that do not consider safety constraints.

As it is seen in Figure 10, the number of additional local maxima is drastically reduced when the normal to cortex component is considered instead of the total current density in Figures 8 and 9. Even in case of the “opposite” configuration, when current streamlines are getting crowded near the sink cluster, the probability is low that they will cross the cortex patches preferentially along the cortical normal vectors.

### Focality

The focality of targeting is one of the key challenges for TES. Electricity cannot be focused in a conductive medium, but seeks the path of least resistance. Nonetheless, focal targeting is often necessary to manipulate one brain area and not affect the others. By simple inspection of Figures 8 and 9, and assuming an upper threshold, the highest focality for T1 is obtained with the LS, “one source,” “opposite,” and ROADSS methods, with a clear high spot concentrated at the target. For T2 and T4, the LS, LCMV, “opposite,” and ROADSS methods are likely the most focal ones, and for T3, all algorithms show the similar focality (except for the “opposite” configuration, where the TE is larger). However, when looking at the half width at half maximum metric (Figure 13A), the highest focality is obtained with the “ring” configuration that concentrates sources and sinks in a smaller region, followed by ROADSS in the second tier, and finally by the LS, LCMV, “one source,” and “opposite” methods in the lower tier. This means that the current density is lower with the ROADSS and “ring” methods in brain regions far from the target. Again, it is worth to note that the similar or even better focality parameters may be achieved with the reciprocity methods, even though they consider at least ten electrodes for sources and sinks, which is not enforced for the LS and LCMV versions used in this work. Note that the local focality (F_LOC_) in Figure 13B is similar for all different methods but it depends more on the targets. For example, T1 can be targeted more focally with all the methods than other targets. This result suggests that the local focality might be mostly determined by the local geometry at the target, i.e., the local bends of the cortex and the local current paths in the CSF near the ROI. This same rationale applies for the local TE metric, seen on Figure 12B. The use of 256 instead of 128 electrodes also increases global focality, this is also observed when comparing Figures 8 and 9. Lastly, the focality is significantly improved when it is defined in terms of the normal to cortex component, as can be deduced from Figure 10. This figure is relevant in the general context of TES as it reveals the great potential of tDCS and TES to produce a very accurate and focal targeting from scalp.

### Modeling Effects

The maximum current density values on the cortex obtained in this study are smaller than in other studies (37, 77), even when the difference in total injected current level (1 versus 2 mA) is factored in. This may be explained in part by the different injection patterns (the ring like pattern versus the opposite one). Also important is the joint effect of the lower skull conductivity (0.008 versus 0.01 S/m) and the higher CSF conductivity (1.79 versus 1.65 S/m) assumed in our study, producing a stronger electrical shield for currents injected from the scalp and correspondingly less current reaching the brain. These differences together with Figure S3 in Supplementary Material emphasize that to correctly assess the therapeutic dose for TES, it is critical to estimate the electrical conductivity of the skull accurately, such as with bEIT. The recent advances in parametric bounded EIT (29–31, 33) allow accurate assessment of scalp and skull conductivities quickly and easily on a routine basis.

Although the unintended current flow was not a major focus of this study, a close look at Figures 8 and 9 shows some unexpected high current density “hot spots” in deep regions. In Figure S3 in Supplementary Material, for example, examining the middle plane between the cerebral hemispheres, the narrow high conductivity CSF paths concentrate current streamlines in a very small region. Similar effects were also observed in previous studies (25, 26). These unexpected and to some extent unintuitive non-target effects emphasize the importance of detailed subject specific models to estimate the complex flow and distribution of the current inside the head.

### Limitations

In real scenarios, the upper bound of injected current density per electrode might be exceeded at the edges of the electrodes because current density tends to be higher on the perimeter of electrodes depending on the contact impedance (38, 59). Also, if considering the use of only one physical current source, the contact impedance at each electrode will determine the injected current distribution, and the upper bound will be exceeded in the channels with lower contact impedance. A detailed analysis of the maximum current densities on scalp, considering different electrode geometries, and multiple electrode contact impedances is out of the scope of this work.

The individual head modeling becomes especially important in the design of TES experiments to predict absolute neurostimulation doses. Therefore, the use of only one head model might be a limitation of the experimental work to normalize a dose across the heterogeneous subject pool. However, in this study, we perform a general theoretical analysis of the reciprocity methods, and a comparative numerical analysis of different mathematical targeting algorithms (the reciprocity methods versus the LS and LCMV methods under the same model conditions, so the simulation results are relative to these other methods). Therefore, we believe that the conclusions derived from our theoretical and numerical results are independent of the head models.

In this work, we have focused on relatively shallow cortical targets near the skull, leaving the analysis of deep targets for future simulations. However, the theoretical analysis of the reciprocity principle explained in Section “Reciprocity” is still valid and thus, it can be used as a guideline for targeting with a focus on optimizing directionality. We expect that due to the nature of the diffusion equation governing the volume conduction and the complexity of the internal conductivity distribution, the intensity, the TE and the focality would be worse for deep targets than for the targets studied in this work, independently of the targeting approach (including the LS or LCMV methods).

Another limitation is that our results are only based on modeling and simulations. We plan to analyze real data experiments to validate our results in practice.

### Reciprocity Principle Targeting using Real EEG Measurements

In this application, it is assumed that there is no head model for a given subject, but there is a real EEG recording, say, from an epilepsy patient or an ERP experiment. Then, one can obtain the poles of the experimental scalp topography online or offline in the scalp space. The question is whether it would be possible to inject the tDCS or TES stimuli into these experimental EEG poles to target (stimulate) exactly the same cortical source (or sources) generating this topography on the scalp. Our analysis shows that if there is one predominant and focal source, the above procedure should theoretically work in terms of delivering the maximum possible current density to the cortical source responsible for this particular topography at a specific instant of time. Ideally, this is the case for focal epilepsy sources or ERPs showing simple topologies. It gets more complicated in a case of distributed or multiple cortex sources generating EEG simultaneously. Anyway, without a model, the current density delivered to other cortical areas remains uncertain. Also, any noise and artifacts in the data could blur the topography pole locations and inevitably make the targeting less precise. Another limitation of using reciprocal TES based on the realistic EEG only is that not all brain ROIs (such as subcortical, mid-brain, or thalamus areas) do produce EEG, but only cortical regions. The reciprocity approach, when the subject specific head model is available, allows to create in simulation virtual EEG on the scalp produced by an artificial electrical source placed anywhere inside the head.

Overall, we believe this is a direct and very practical application of targeting based on reciprocity that should be carefully studied as it might have a great impact in clinical applications.

### Impacts on Clinical Applications of TEN in Psychiatric Disorders

Multiple studies in medicine and neuroscience employing various experimental approaches in humans, such as functional imaging, lesion method, and invasive brain stimulation aligned with psychometrics score techniques, have provided evidence of depression pathology in the brain. These research point to the ventromedial and dorsolateral areas of the prefrontal cortex and associated networks as critical neural sources correlated with clinical symptoms of depression (78). Over the last few decades, several non-invasive brain stimulation methods such as Electrical Convulsian Therapy (ECT) (79), repetitive TMS (80), cranial electrotherapy stimulation (CES), which is similar to tACS, and tDCS have been established or reintroduced as options of treatment for depression, anxiety, fatigue, and chronic pain (81). All these methods are not free of limitations. ECT stimulates the entire frontal cortex indiscriminately, requires general anesthesia, muscular relaxation, and induction of a seizure, it involves side effects such as memory disturbances, and it is usually used only for treatment of severe depression symptoms in drug refractive patients. The efficacy of rTMS and tACS/tDCS has been assessed in a number of recent studies [see the reviews in Ref. (5, 82)] but results show high individual variability in response, generating debates on the clinical effectiveness of TES (13–15). Refining the anatomical target based on functional imaging, such as fMRI or fEEG, to customize electrode placement derived from individual anatomy may dramatically improve results. Currently, the most frequently used “standard” procedure of coil positioning in rTMS depression treatment locates dorsolateral prefrontal cortex in line with the simplistic fit-for-all 5 cm rule (5 cm anterior to the “hand motor hotspot”) and has been shown to be inferior to the “neuronavigated” procedure (83). The placement of the tDCS/tACS stimulating patches is also done in the “standard” scalp coordinates defined by 10–20 EEG electrode system (81, 84). We believe that this standard placement is a major limitation because current paths are estimated in the scalp coordinate space, but the spatial relation between electrode on the scalp and underlying cortex has a lot of individual variability and does not ensure that current will be optimally delivered to the intended cortical target. Given an MRI-based computational head model of a patient, the integration of the reciprocity-based targeting techniques described in this paper with dense array EEG source localization will change the current practice of clinical application of TES by providing in real time a cortical ROI associated with a psychiatric disorder and the optimal electrode pattern for its stimulation precomputed in the high resolution individual head model space.

## Conclusion

In this work, it was shown theoretically and by means of simulations that the reciprocity principle empirically extended to include the safety constraints can be successfully used as an optimization strategy for designing current injection patterns in tDCS or TES to stimulate specific brain regions. This design is based on the computation of the EEG fields of a single dipole oriented as the desired target, thus being computationally much less expensive than other traditional methods such as LS, LCMV, or simulated annealing. Moreover, both the current injection solutions and the imprinted on cortex current density maps are very similar to the non-iterative versions of the LS and LCMV algorithms used in this work as the reference standards.

Even with the constraint of an upper bound for the current driven per electrode, the reciprocity methods performed similarly to, or better than, the unconstrained versions of the LS or LCMV methods, and this is a very important result. Note that each method is optimal in some respect, but there are several performance metrics of interest. All analyzed metrics are important and their relevance might depend on the specific application. The LS method is an optimization of the squared error between the desired and possible current densities, so the desired density should be known *a priori* at ROI and beyond. If these conditions are met, it is optimal in that sense. The LCMV method has the hard constraint of directionality, and it is the best one for directionality (as seen in Figure 11C). Equivalently, the reciprocity methods optimize the maximum current density in the desired orientation, and it is the best one in this aspect. Overall, the reciprocity method performs better where it is expected (intensity of the current directional component) and has still comparable or better performance than LS or LCMV in other metrics. Specifically, the reciprocity methods outperformed LS and LCMV in the total intensity metric and global focality, and they performed similarly in the directionality, TE, and local focality metrics. As it could be expected, the “opposite” and “ring” configurations were best suited for tangentially and radially oriented targets, respectively.

In general, the LS/LCMV solutions for a number of significant electrodes involved into the optimal pattern for TES are rather sparse (see Figures S1 and S2 in Supplementary Material). Cutting the dozens low intensity injecting electrodes from the optimal pattern does not change the impressed intensities on the cortical ROI much as it can be seen comparing the LS/LCMV and “one source” reciprocity results in Figures 8 and 9. Yet, this fact should not be misleading with a need for a dense array, as the location of those active injecting electrodes that are left is more precise in a dense array than in a sparse array.

The novel reciprocity-based ROADSS algorithm developed in this work resulted in the current injection patterns, the most similar to the LS and LCMV solutions. It was more robust in terms of TE, global focality, and maximal current density delivered to ROIs with different targeting orientations. However, the ROADSS algorithm did show lower directionality of delivered current. An important question for dosing studies is whether directionality is indeed a major issue in TES. In principle, the primary polarization of cortical neurons will be achieved with surface-normal currents, and the tangential currents should not significantly affect neurons. Only dosing studies will test this assertion. If considering only the normal to cortex component of the current density (Figure 10), tDCS and TES have a very high potential for the precise and accurate targeting. Note the great improvement in focality and TE in this figure compared to Figures 8 and 9, where the total amplitude of the current density is considered. In general, the use of 256 instead of 128 electrodes reduced current density and local maxima far from the targets thus improving focality and, to some extent, current directionality and current density on target.

We believe there is room to improve targeting algorithms based on the reciprocity principle (like ROADSS) in the future research. One of the goals could be to extend the reciprocity targeting technique to the spatially extended and multiple (not necessarily contingent) ROIs and deep targets. For instance, one can imagine the situation when all four targets in this study need to be targeted at the same time. It is very likely that a simple linear combination of the optimal “one source” configuration with the weighted current injection levels will do the job, but it needs validation and comparison with other methods. Another goal would be to improve directionality while maintaining the achieved intensity, the TE, and focality metrics, and also to consider safety and hardware complexity constraints in more detail. For maximum flexibility in targeted dosing, the advantages of independent current sources for each electrode are clear. However, because each source interacts with all sinks, there is a considerable challenge in electronics to balance a dense array of electrodes such that each has a target current level, given that each electrode’s current limit circuit will interact with all others.

What is clear from these and other simulations is that the electrical conductivities of the scalp and skull play major roles in TES current density dose delivery estimation, for both target and non-target current delivery. In order to validate current delivery generally, and to adjust to the properties of the individual’s scalp and skull specifically, it is important to include conductivity estimation methods such as bounded EIT in the planning process for estimating TES current delivery.

## Author Contributions

MF-C contributed to the design and methodological ideas of this work, performed the computational simulations and wrote the initial draft of this paper. ST directed the whole work and wrote several parts of the paper. ST, PL, and DT developed the original ideas of this work. ST and PL collected the MR and CT data. EA contributed to the head model construction. All authors took part in revising the manuscript, approved the final version, and agree to be accountable for all aspects of the work in ensuring that questions related to the accuracy or integrity of any part of the work are appropriately investigated and resolved.

## Conflict of Interest Statement

The commercial use of the reciprocity between measured brain currents on the scalp (EEG) and transcranial neuromodulation (tDCS, tACS, and GTEN) is protected by US Patents No. 6,594,521 and No. 7,840,250. ST, PL, EA, and DT are employees of Electrical Geodesics, Inc., a manufacturer of dense array EEG systems.

## Supplementary Material

Figure S1 **Current injection patterns for the 128 electrode sensor net simulations, showing the electric current injected by each electrode [mA]**. Each row corresponds, from top to bottom, to each of the different trial targets from T1 to T4.

Figure S2 **Current injection patterns for the 256 electrode sensor net simulations, showing the electric current injected by each electrode [mA]**. Each row corresponds, from top to bottom, to each of the different trial targets from T1 to T4.

Figure S3 **Current density coronal slice computed in the finite element model with a single source near the vertex (top) and multiple sinks at all other electrodes (“one-source” configuration in the reciprocity method)**. The gray circles show high current density at narrow CSF regions, and the black circles show regions of the cortex that are strongly stimulated because of their proximity to these high current density CSF regions.

Figure S4 **Current density vs. skull conductivity**. The four lines depict the total current density at the four targets for the reciprocity “one-source” approach using the 128 sensor net.
