# Supplementary figures and images for: Transcranial Electrical Neuromodulation Based on the Reciprocity Principle
Source: Front Psychiatry. 2016 May 27;7:87. doi: 10.3389/fpsyt.2016.00087 (PMC4882341; doi:10.3389/fpsyt.2016.00087)

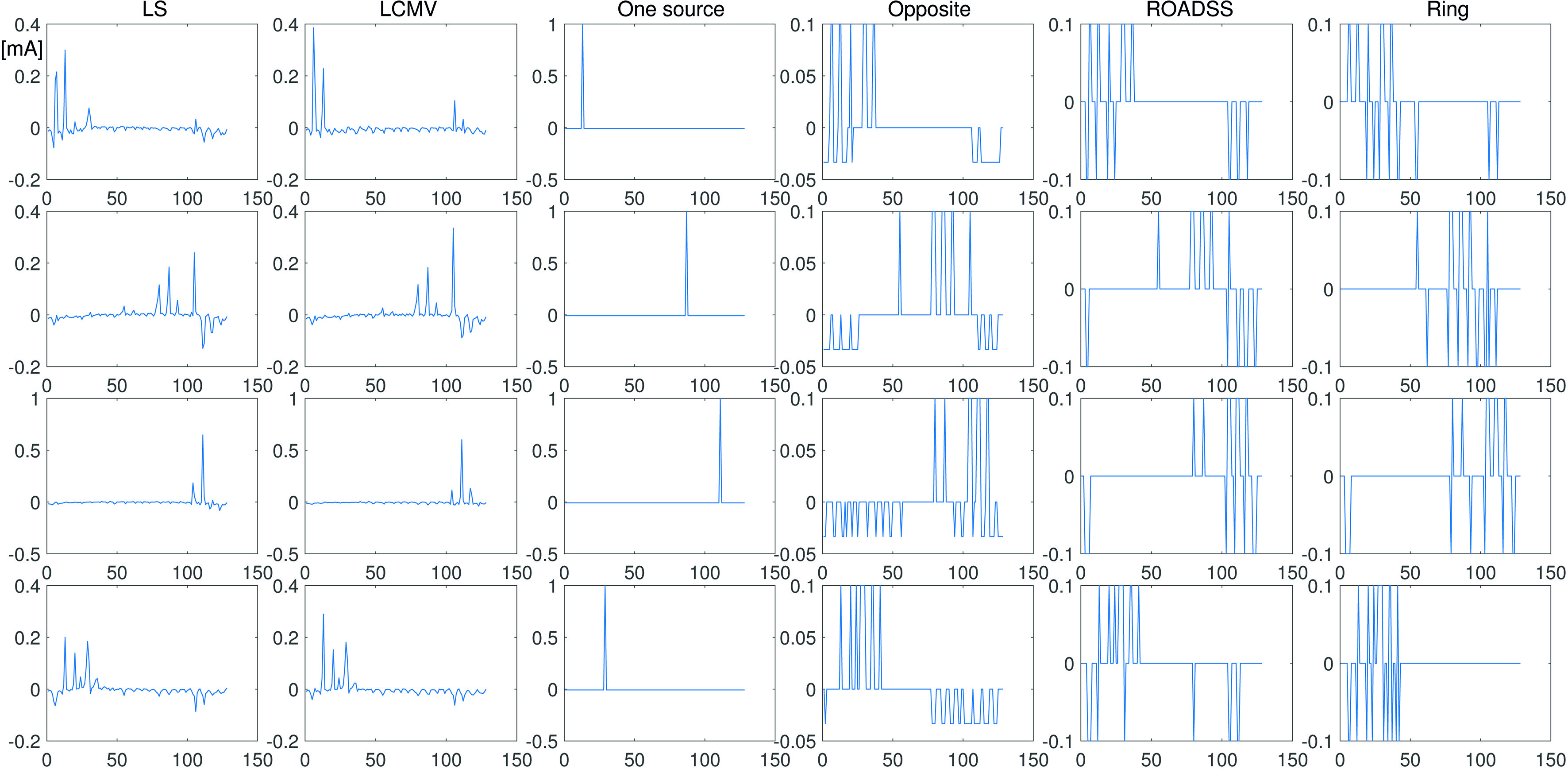

Supplement: Figure S1 — Current injection patterns for the 128 electrode sensor net simulations, showing the electric current injected by each electrode [mA]. Each row corresponds, from top to bottom, to each of the different trial targets from T1 to T4. [file image_1.jpg]

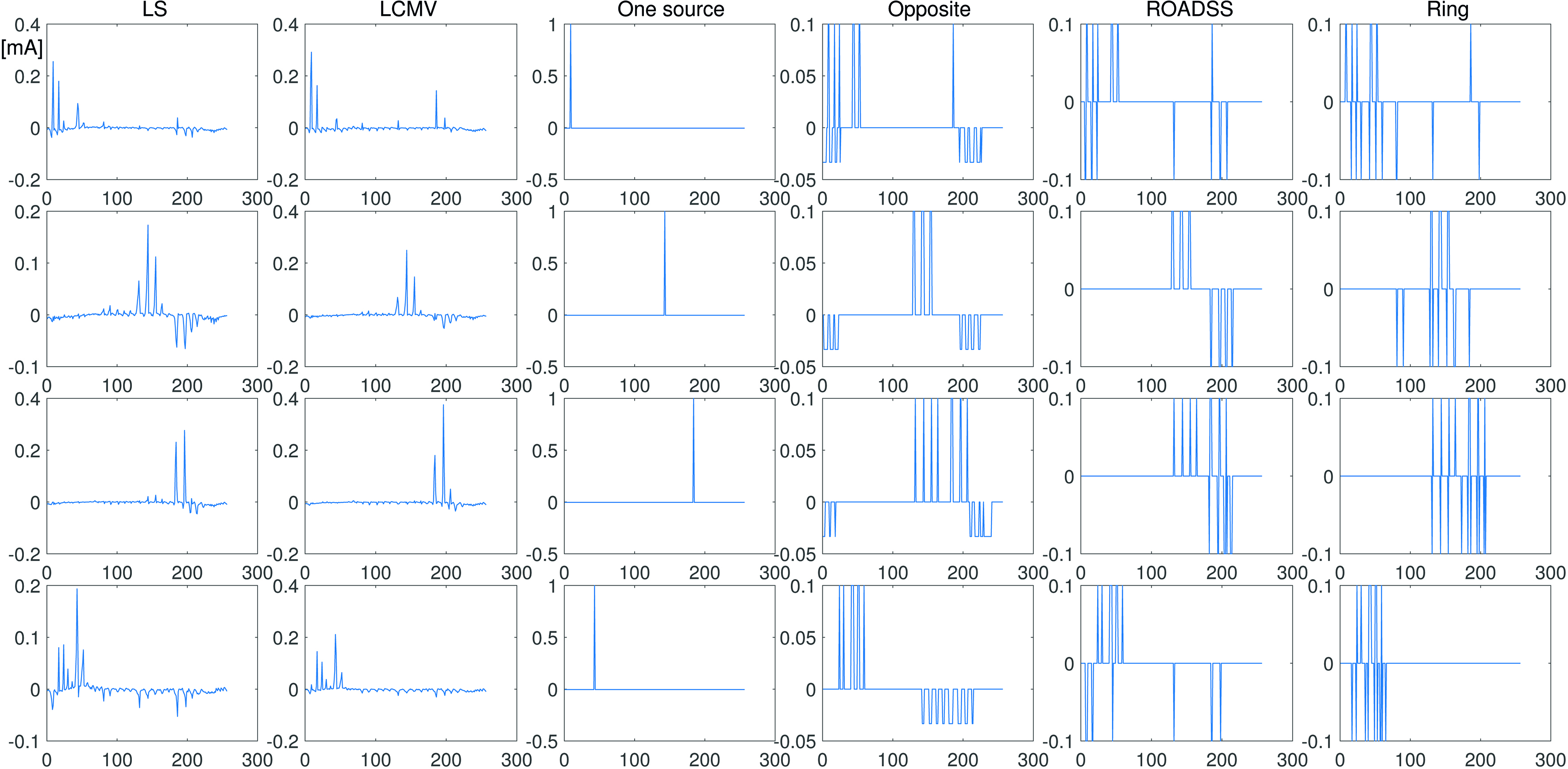

Supplement: Figure S2 — Current injection patterns for the 256 electrode sensor net simulations, showing the electric current injected by each electrode [mA]. Each row corresponds, from top to bottom, to each of the different trial targets from T1 to T4. [file image_2.jpg]

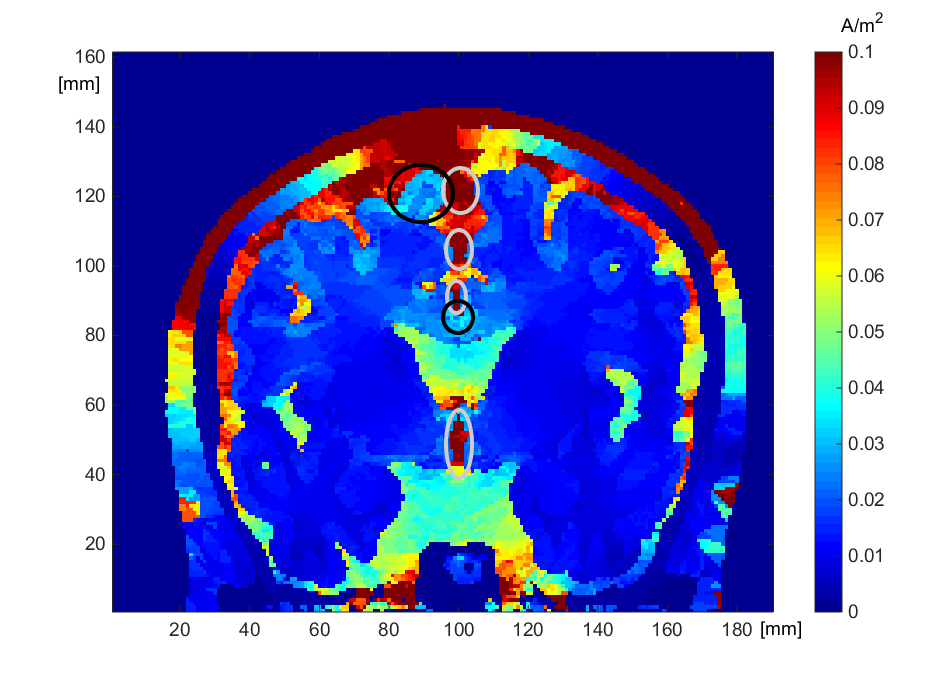

Supplement: Figure S3 — Current density coronal slice computed in the finite element model with a single source near the vertex (top) and multiple sinks at all other electrodes (“one-source” configuration in the reciprocity method). The gray circles show high current density at narrow CSF regions, and the black circles show regions of the cortex that are strongly stimulated because of their proximity to these high current density CSF regions. [file image_3.jpg]

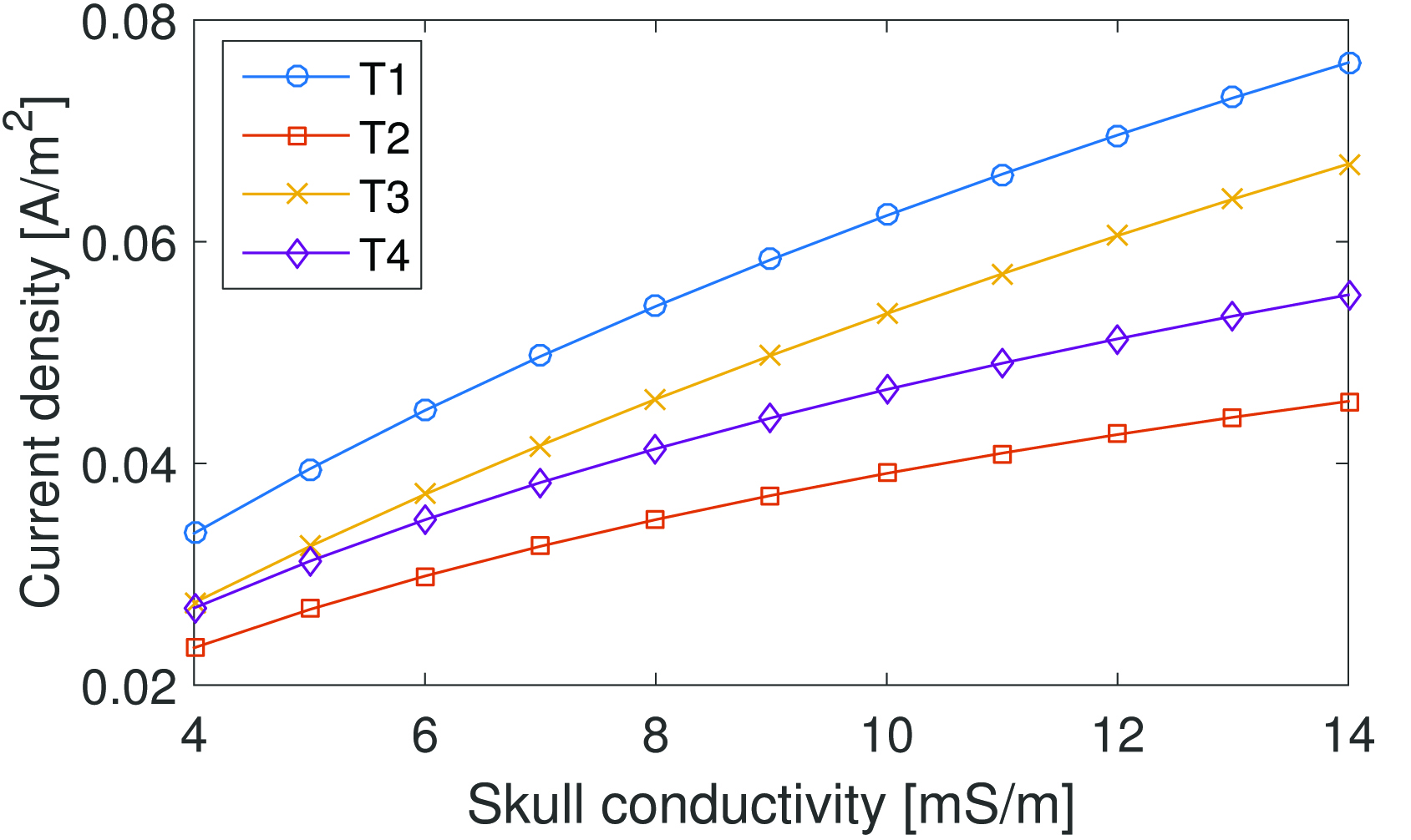

Supplement: Figure S4 — Current density vs. skull conductivity. The four lines depict the total current density at the four targets for the reciprocity “one-source” approach using the 128 sensor net. [file image_4.jpg]
